# Effects of a New Type of Shrinkage-Reducing Agent on Concrete Properties

**DOI:** 10.3390/ma13133018

**Published:** 2020-07-06

**Authors:** Mari Masanaga, Tsuyoshi Hirata, Hirokatsu Kawakami, Yuka Morinaga, Toyoharu Nawa, Yogarajah Elakneswaran

**Affiliations:** 1 Ethylene Oxide (EO) Research and Development Department, NIPPON SHOKUBAI, Osaka 564-0034, Japan; mari_masanaga@shokubai.co.jp (M.M.); t_hirata56@me.com (T.H.); 2 Division of Sustainable Resources Engineering, Faculty of Engineering, Hokkaido University, Kita 13, Nishi 8, Kita-ku, Sapporo 060-8628, Japan; morinaga@eng.hokudai.ac.jp (Y.M.); nawa@eng.hokudai.ac.jp (T.N.); elakneswaran@eng.hokudai.ac.jp (Y.E.)

**Keywords:** shrinkage-reducing agent, compressive strength, splitting tensile strength, freezing and thawing, spacing factor

## Abstract

Shrinkage-reducing agents have been developed to mitigate shrinkage and to control cracks in concrete. This study aims to evaluate the impact of a newly developed shrinkage-reducing agent (N-SRA) on concrete properties and to compare its properties with a conventional shrinkage-reducing agent (C-SRA). The hydration rate, compressive strength, splitting tensile strength, shrinkage, occurrence of cracking, and freezing and thawing were investigated. N-SRA showed higher surface tension than C-SRA and reduced shrinkage to the same degree as C-SRA with half the dosage of C-SRA. The addition of N-SRA or C-SRA did not influence the early compressive strength but slightly reduced splitting tensile strength at seven days. Concrete with N-SRA showed higher compressive strength at 28 days than those of concrete with C-SRA or without SRA. Furthermore, concrete with N-SRA extended the period for the occurrence of shrinkage cracking under restrained conditions. It was found that N-SRA provided excellent freezing and thawing resistance because of the formation of good air voids, while C-SRA demonstrated inefficient behaviour in such an environment.

## 1. Introduction

Shrinkage is a common problem in concrete, when it is not properly handled. Shrinkage causes cracking, accelerates ingress of deleterious ions (chloride, sulphate, etc.), and eventually leads to failure, thereby shortening the service life of concrete structures. A possible way to mitigate shrinkage-induced cracking in concrete is to add a special type of organic chemical admixture called shrinkage-reducing agent (SRA) to the concrete mixture. The practice of using SRA, a class of surfactants, was initiated in Japan in the 1980s and is now available worldwide [1]. Conventional SRA is a polyoxyalkylene with or without a hydrophobic group at the end (e.g., a blend of propylene glycol derivatives [2], 2-butoxy ethanol with four ethylene oxide adduct [3]), and forms micelles in aqueous solution due to amphiphilic molecules [4,5]. Various studies have been conducted on the performance of conventional SRAs for shrinkage reduction [2,3,4,5,6,7,8,9,10,11,12,13].

When the water in hardened concrete evaporates, a meniscus is formed at the air–solution interface of the capillary pore when the surrounding humidity is lower. Consequently, the surface tension in the meniscus pulls the pore walls inward causing shrinkage in the concrete. However, the addition of conventional SRA to the concrete mixing water lowers the surface tension (to approximately 40 dynes/cm) of the pore solution in the hardened concrete, resulting in a reduction in the drying shrinkage. The conventional SRA remains in the pore system even after the concrete hardens, and it continues to reduce the surface tension effect that contributes to drying shrinkage. It has been reported that, in addition to reducing the pore solution surface tension, the conventional SRA has several other beneficial effects [5,14,15,16]. Conventional SRA helps to reduce the capillary stress formed by autogenous shrinkage and the associated restrained shrinkage cracking in high-strength concrete with low water-to-cement ratio [5]. The conventional SRA shows a lower weight loss in concrete, indicating the prevention of water evaporation. Moreover, conventional SRA exhibits the absence of autogenous shrinkage [6] and resistance to restrained shrinkage cracking, through extended time to cracking [2] and shallower crack width [8,17], when compared to concrete without SRA. It has also been reported that SRA significantly improves durability by reducing sorptivity and wetting moisture diffusivity [16] and increasing the pore solution viscosity, which can reduce the diffusion coefficient of ions [15]. Nevertheless, conventional SRA also shows some negative effects, such as a slight decrease in the early age compressive and flexural strengths [5,6,7,8], a delay in setting [5,6,17,18], and freezing and thawing damage depending on the air void system [19].

Although most studies suggest that lowering the surface tension of the pore solution is the main mechanism for the shrinkage reduction by conventional SRA [2,3,4,5,6,7,8,9], recent studies have shown that the shrinkage-reduction mechanism of SRA is very complicated, and that lowering the surface tension is not the only reason for shrinkage reduction [9,16,20]. An increasing number of studies have focused on the development of new types of SRA to overcome the negative effect of conventional SRA and to enhance its performance and economic efficiency. Therefore, in this study, a newly developed shrinkage-reducing agent (N-SRA) was introduced, and its properties and performance were compared with those of a conventional SRA. Finally, the acting mechanism of N-SRA is discussed.

## 2. Experimental

### 2.1. Materials

Ordinary Portland cement (OPC) manufactured by Taiheiyo cement corporation was used in this study. The chemical composition and physical properties of OPC are given in Table 1.

N-SRA is a newly developed, completely water-soluble polymer consisting of hydrophilic monomers with an optimal molecular weight. The design concept of N-SRA itself does not form a micelle in the pore solution of hydrated cement. A commercial polyoxyalkylene-type SRA called C-SRA was used for comparison. C-SRA is five-molar ethylene oxide and one-molar propylene oxide adduct to a methyl alcohol based on ^1^H NMR analysis. Generally, the structure of C-SRA, which has hydrophilic group as well as hydrophobic group, is called amphiphilicity, which is widely used as a surfactant in industry.

The hydrodynamic radius (R_H_) was determined by dynamic light scattering (DLS) method using a Zetasizer Nano ZSP (Malvern Instruments Ltd., Malvern, UK). The hydrodynamic radius was calculated by the Stokes-Einstein equation, which defines the relationship between the hydrodynamic radius of a sample and its diffusion rate due to Brownian motion. The sample concentration was 0.5 wt.% in the solution, and the measurement temperature was set to 25 °C. The pore solution of hydrated cement was synthesized by dissolving the following amount of chemicals: 1.72 g/L CaSO_4_: 2H_2_O, 6.959 g/L Na_2_SO_4_, 4.757 g/L K_2_SO_4_, and 7.12 g/L KOH (pH = 13.1). The determined R_H_ in the simulated pore solution was 9 nm and 459 nm for N-SRA and C-SRA, respectively. The results suggest that N-SRA is sufficiently dissolved. On the other hand, C-SRA is agglomerated and formed micelles due to hydrophobic association via the salting-out effect.

### 2.2. Casting and Testing of Concrete

**Figure 1:**
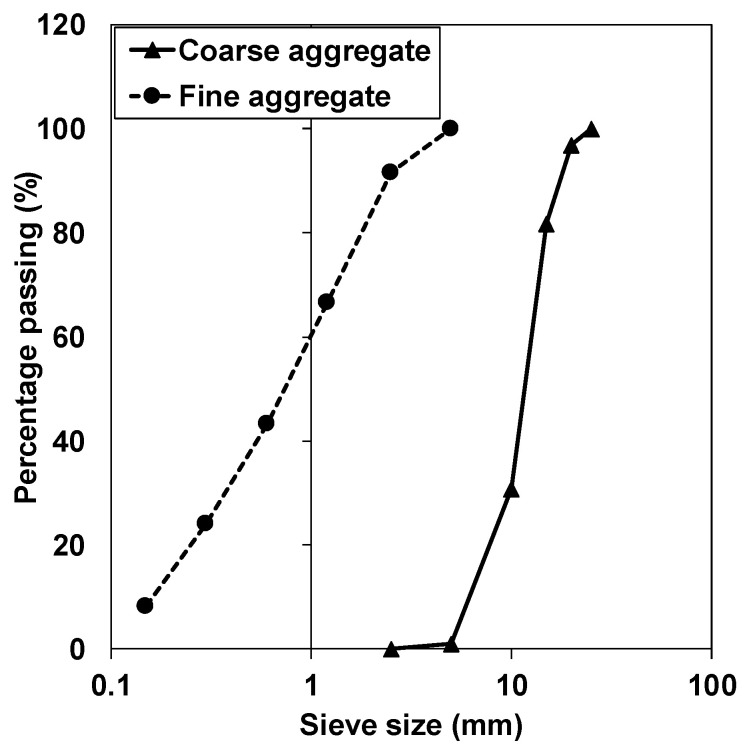
Grading curves for fine and coarse aggregates.

The concrete mixture proportions and properties of the fresh concrete are summarised in Table 2. The starting materials were selected in accordance with JIS A 5308, and the concrete formulations were determined as reference to JIS A 6204. Figure 1 shows the aggregate grading curves used in the preparation of concrete. Concrete made with C-SRA and N-SRA are denoted as Concrete-C and Concrete-N, respectively. Concrete without SRA is used as the reference. The SRA dosage was 2% and 1% by weight of cement (% by weight of cement (BWOC)) for C-SRA and N-SRA, respectively. The concrete was cast with a dual-axle revolving-paddle mixer with a capacity of 50 L. First, cement, fine aggregates, and coarse aggregates were mixed without water for 10s. Second, water with superplasiticiser (SP), Air-entraining (AE) agent, defoamer, and N-SRA or C-SRA were added, and mixed for 1 min. Mixing was then stopped to scrape the mortar off the mixer wall, followed by another mixing for 1 min. A polycarboxylate ether type superplasticizer (MasterGlenium SP8SV, BASF, Tokyo, Japan) was used as the SP, and its dosage was adjusted to obtain the target slump value of 18 ± 2 cm. It should be noted that the SP dosage for the concrete with N-SRA was lower than that in the concrete with C-SRA because N-SRA has a slight water-reducing effect. The air content was controlled to the range of 4.5 to 5.5 vol.% by using both AE agent and defoamer. Both coarse and fine aggregates were used in the saturated surface-dry condition. The amounts of SP, SRA, AE agent, and defoamer were subtracted from the amount of mixing water.

The workability of concrete and the air void content in concrete were determined in accordance with Japanese Industrial Standard JIS A 1101 (the slump test) and JIS A 1118 (air content: a pressure method), respectively. The air void characteristics of concrete were also identified using an air void analyser (AVA, Germann Instrument, Copenhagen, Denmark) [21]. The size distribution of air bubbles can be measured through the buoyancy changes of the air bubbles by Stokes’ law. Initial and final setting times were determined in accordance with JIS A 1147. The fresh concrete was cast into the formworks, covered by plastic sheeting, and stored at 20 °C until further experiments.

### 2.3. Measurement of Hardened Concrete Properties

**Figure 2:**
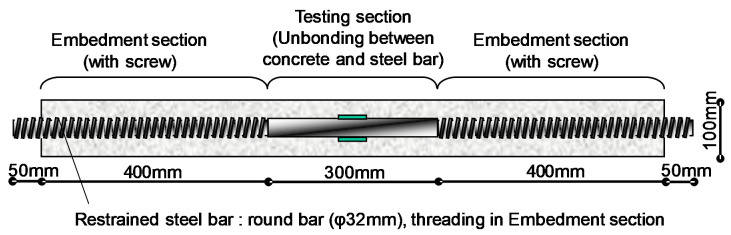
Schematic diagram of specimen for restrained shrinkage cracking test.

Standard test methods were adopted to determine the hardened concrete properties such as free shrinkage strain (JCI-TC083A, formwork: JIS A 1132), compressive strength (JIS A 1108, formwork: JIS A 1132), splitting tensile strength (JIS A 1113, formwork: JIS A 1132), and freeze–thaw resistance (JIS A 1148, formwork JIS A 1132). To measure the free shrinkage strain, a strain gauge (PLT-60-5LT, Tokyo Sokki Kenkyujo Co., Ltd. Tokyo, Japan) was placed inside the formwork. Restrained shrinkage cracking test was conducted according to the shrinkage crack evaluation test method for concrete, which was proposed by the Japan Concrete Institute (Figure 2).

### 2.4. Cement Hydration

The heat generated during cement hydration was quantified using a conduction calorimeter (TAM Air, TA Instruments, New Castle, UK) at 20 °C. A cement paste with a water-to-cement ratio (w/c) of 0.5 and N-SRA dosage of 1% BWOC was used for the measurement.

For the XRD-Rietveld analysis, cement paste with w/c ratio of 0.5 was prepared using Hobart mixer N50. Initially, water was added to cement, and mixed for 30 s. Mixing was then stopped to scrape cement paste off the wall, followed by another mixing for 3 min. N-SRA was added to the mixing water and its amount was subtracted from the amount of mixing water. The prepared cement slurry was stored in a plastic bottle and agitated with a spatula at intervals of 1 h for 7 h to prevent bleeding. The cement slurry was poured into a settled formwork (φ 50 mm × height 10 mm) and sealed with a plastic sheet and then stored for 24 h at 20 °C. Afterwards, it was demoulded and cured in water. Specimens were taken after three and nine weeks of hydration and were broken into pieces using a hammer. Five grams of the obtained powder sample was mixed with 50 g acetone for 24 h to stop the hydration. After acetone was completely removed by suction filtration, the samples were stored at 40 °C for 6 h before the hydration rate was measured.

The XRD patterns were analysed using a Rigaku MultiFlex X-ray generator (Tokyo, Japan) with CuKα radiation ranging from 5° to 70° (2θ) at a scanning rate of 6.5° (2θ) per min and a step size of 0.02° (2θ). In addition to the qualitative phase analysis by XRD, the Siroquant-Rietveld program was used to quantify the phases generated in the solids. The quantitative phase analysis by XRD is abbreviated as Q-XRD. Based on Q-XRD and the measurements of weight loss on ignition, the weight of HCP in percentage can be converted to mass in grams. Loss on ignition was conducted by heating reacted solids to 950 °C for 2 h, and the weight loss of the solids due to heating was calculated. Hydration degree of hydrated cement by XRD was calculated according to [22].

## 3. Results

### 3.1. Surface Tension of SRA

**Figure 3:**
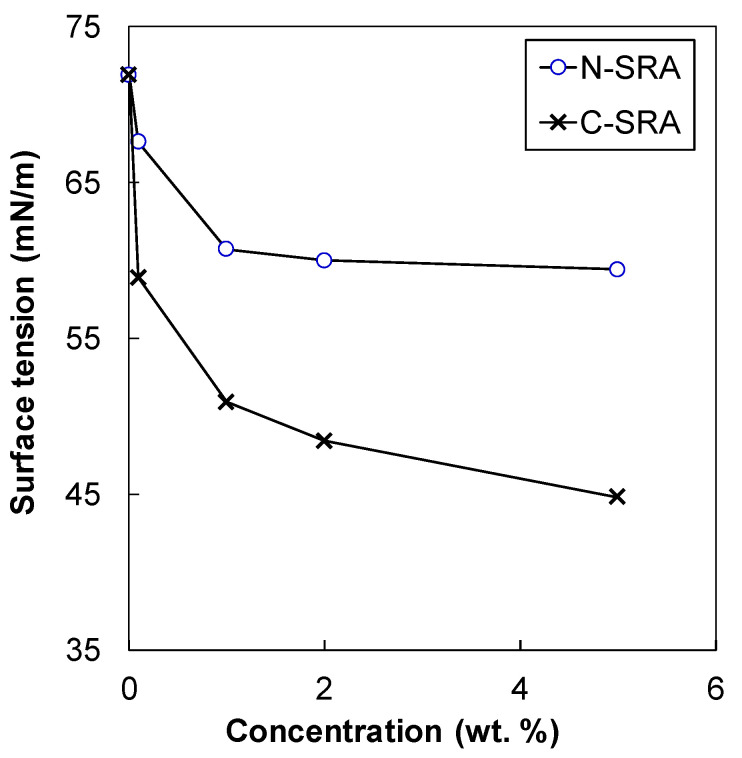
Surface tension of newly developed (N-SRA) and conventional (C-SRA) shrinkage-reducing agent in deionic water.

The hydrophile-lipophile balance (*HLB*) value was calculated according to Griffin’s method [23]:

*HLB* = 20 × *M_h_/M* (1)

where *M_h_* is the molecular mass of the hydrophilic portion of the molecule and *M* is the molecular mass of the total molecule. The calculated HLB values for N-SRA and C-SRA were 18.9 and 14.2, respectively. Therefore, N-SRA is more hydrophilic, which does not lower the surface tension in the aqueous solution. To confirm this, the surface tension of the liquid phase was determined using a Lauda tensiometer. The measurements were conducted at 0.01, 0.05, 0.5, 1.0 and 5.0 wt.% aqueous solutions of SRAs at 20 ± 3 °C by the du Nouy method using a platinum ring. The experimental results are shown in Figure 3. The obtained results for C-SRA are consistent with the results reported in the literature [5,9]. Furthermore, the surface tension of N-SRA is higher than that of C-SRA, which agrees with the theoretical prediction.

### 3.2. Concrete Properties

**Figure 4:**
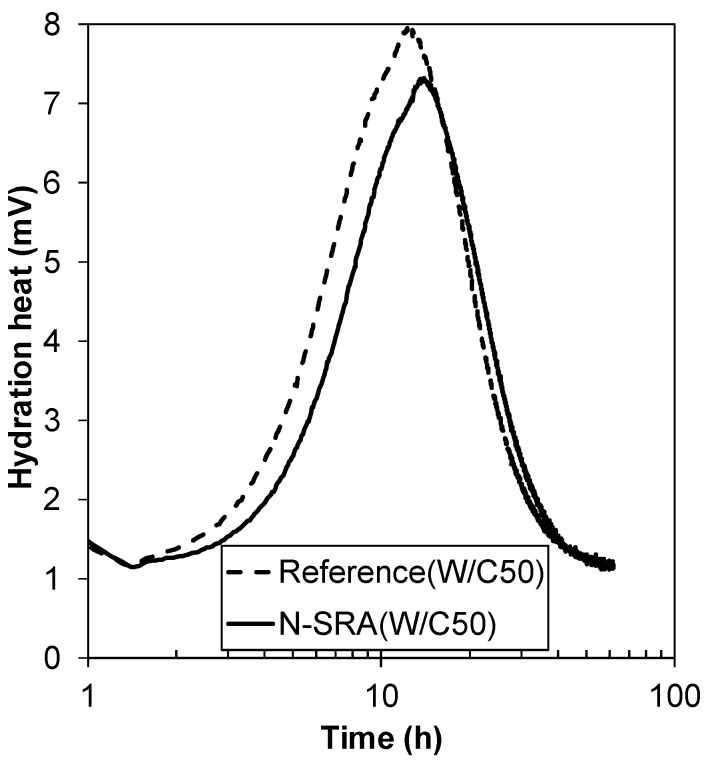
Calorimetric curve during the initial cement hydration.

**Figure 5:**
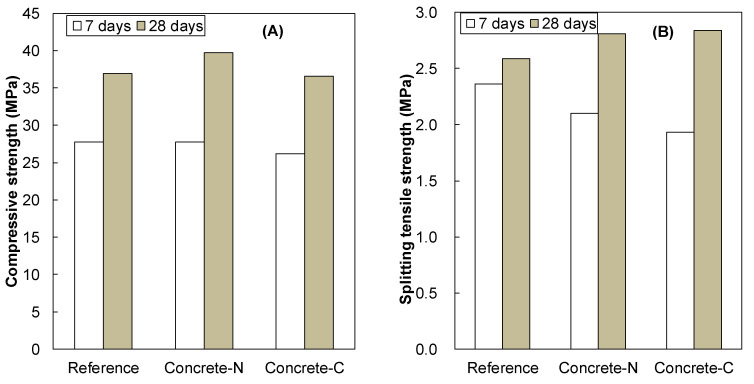
(**A**) Compressive strength and (**B**) splitting tensile strength of concrete with N-SRA and C-SRA compared with reference.

The measured setting times are tabulated in Table 2. There was no significant difference between N-SRA and C-SRA, indicating that both SRAs slightly delay the setting time by 45 min to 1 h (see Table 2). The initial heat evolution of cement hydration with N-SRA is slower than that of the reference (see Figure 4), which is attributed to the delay in setting time of N-SRA. The compressive strength and spilling tensile strength of concrete consisting of SRA were compared with those of the reference concrete in Figure 5. This demonstrates that the strength continues to develop with hydration. The results showed that the addition of SRA does not affect the early compressive strength of concrete, but it slightly reduces early splitting tensile strength. Furthermore, concrete with N-SRA shows somewhat higher compressive strength of concrete at 28 days in comparison to the concrete with C-SRA or reference concrete.

**Figure 6:**
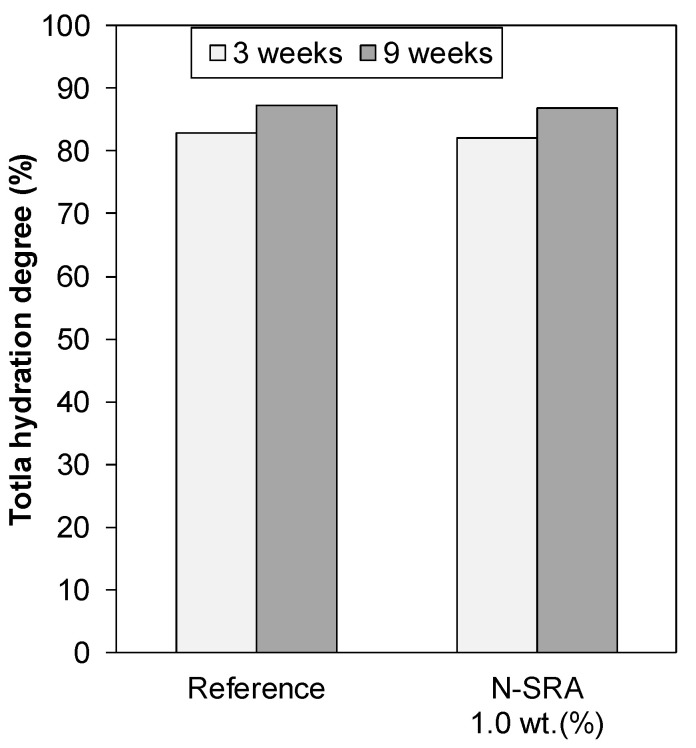
Total hydration degree of cement paste hydrated for three and nine weeks with and without N-SRA.

In order to understand the strength development behaviours of the samples, the total hydration degree of cement in the cement pastes with N-SRA and without SRA at curing periods of three and nine weeks was determined by XRD analysis. The results are shown in Figure 6. The hydration degree of cement for the concrete with N-SRA was almost the same as that of the reference until nine weeks. This suggests concrete with N-SRA achieved approximately the same later-age concrete strength (even after 28 days) as the concrete without SRA.

### 3.3. Concrete Shrinkage

**Figure 7:**
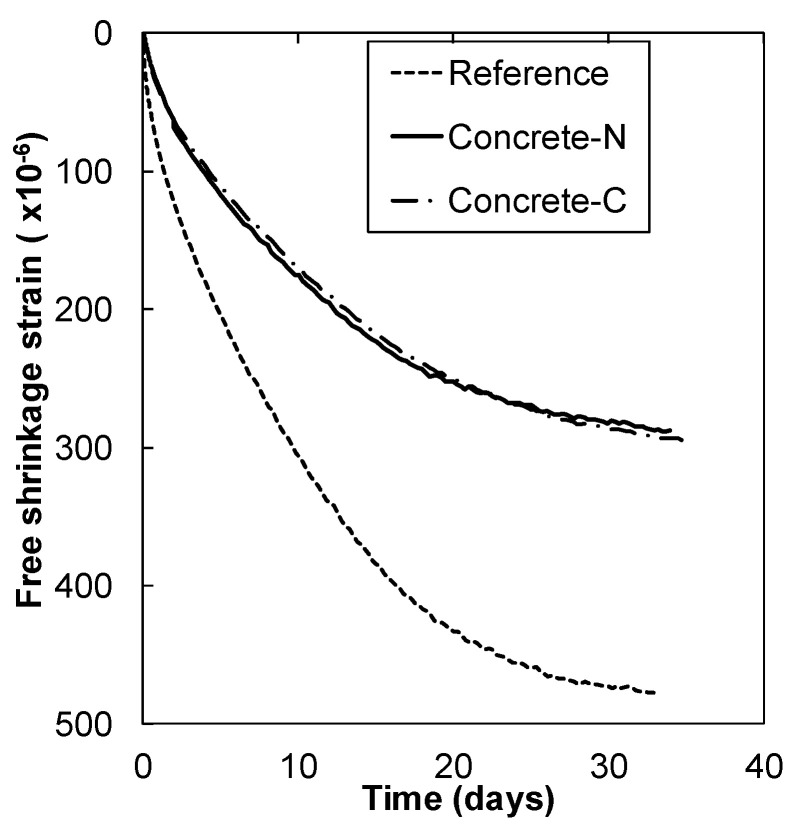
Free shrinkage strain of N-SRA and C-SRA.

**Figure 8:**
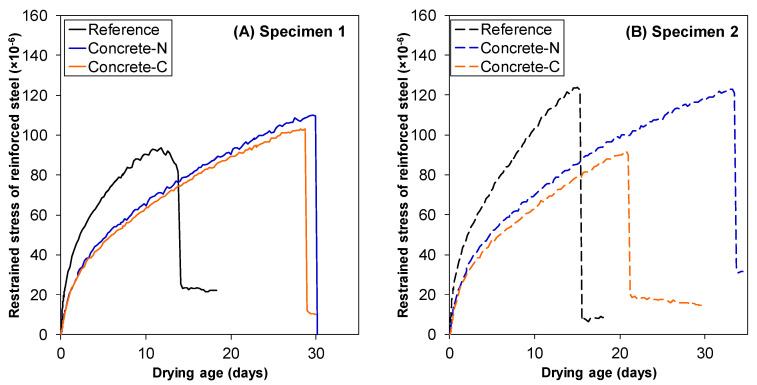
Relationships of restrained stress of reinforced steel and drying age. (**A**) Specimen 1 (**B**) Specimen 2.

**Figure 9:**
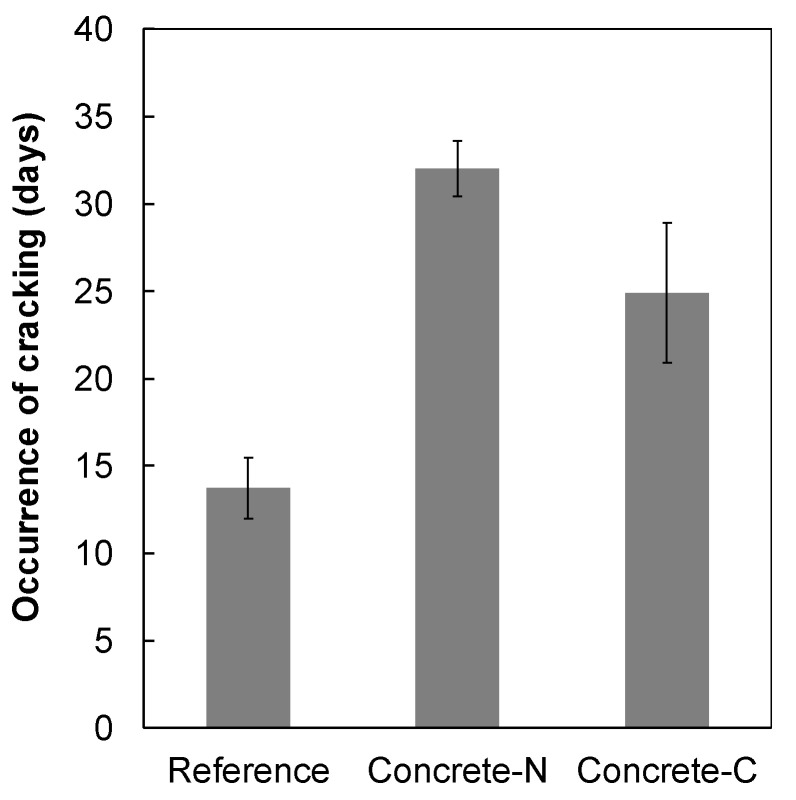
Resistance to restrained shrinkage cracking for concrete with N-SRA or C-SRA.

The free shrinkage strain of concrete with N-SRA (Concrete-N) and C-SRA (Concrete-C) as a function of time are shown in Figure 7. Inclusion of SRA reduced the shrinkage by approximately 50% compared to that of the reference. Both N-SRA and C-SRA showed the same degree of shrinkage reduction, despite the fact that N-SRA required only half the dosage of C-SRA. However, N-SRA was ineffective at reducing surface tension (Figure 3). Figure 8 shows the restrained stress of the reinforced steel in the concrete specimen as a function of the drying age. The results were obtained for two specimens in each case. The restrained stress of concrete also increases with that of the reinforced steel bar, and concrete cracking occurs when the restrained stress exceeds a specific fracture criterion such as the tensile strength of concrete. When compared to the others, concrete with N-SRA resists cracking for a longer period. Based on the results shown in Figure 8, the time required for the concrete to crack was determined, and the impact of SRAs on the occurrence of shrinkage cracking is shown in Figure 9. The cracking time was the average of two measurements. The determined cracking time was 13.8 days for reference, 32.0 days for Concrete-N, and 24.9 days for Concrete-C, indicating that N-SRA reduces the shrinkage cracking of restrained concrete.

The restrained shrinkage stress of concrete when cracking occurred was calculated from the restrained stress of reinforced steel as follows:

*σ_c_* = (*E_s_* × *ε_s_* × *A*_s_)/*A*_c_ (2)

where *σ_c_* is the restrained stress of concrete when cracking occurs (MPa), *E_s_* is the elastic modulus of restrained steel (MPa), *ε_s_* is the restrained stress of reinforced steel when cracking occurs, *A*_s_ is the area of restrained steel (mm^2^), and *A*_c_ is the area of the concrete specimen (mm^2^).

The calculated restrained stresses when cracking occurred were 2.0, 2.2, and 1.8 MPa for the reference concrete, Concrete-N, and Concrete-C, respectively. This indicates that the fracture criterion of cracking is almost the same despite the presence or absence of SRA.

### 3.4. Resistance to Freezing and Thawing Action

**Figure 10:**
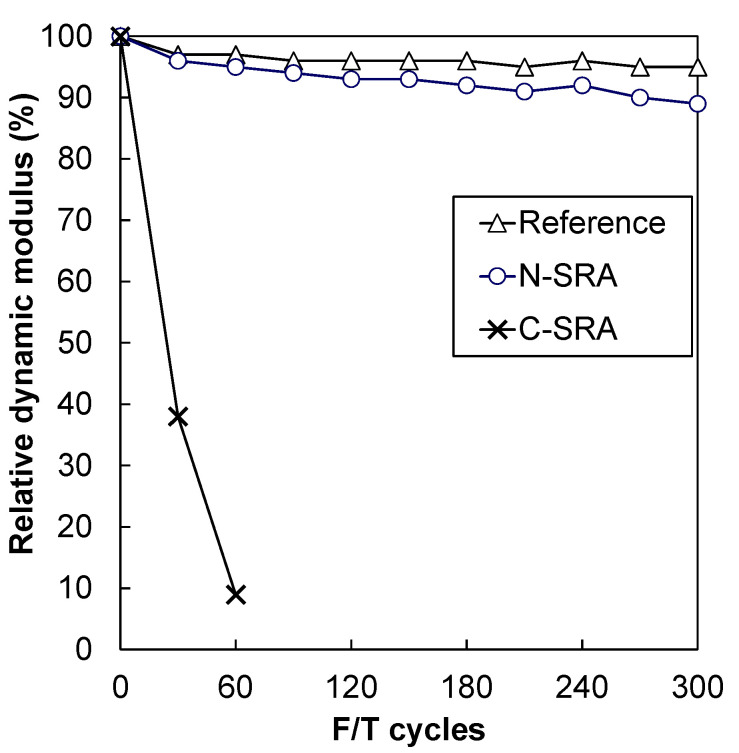
Freezing and thawing test results.

The results of the freezing and thawing tests are shown in Figure 10. The relative dynamic modulus of Concrete-N was maintained above 90%, which was similar to that of the reference concrete. However, as soon as testing started, the relative dynamics modulus of Concrete-C decreased rapidly. It should be emphasized that Concrete-N adequately meets the requirements for the specification of chemical admixture for concrete in accordance with Japanese Industrial Standard JIS R 6204: the relative dynamic modulus should be beyond 60% at 200 cycles of freezing and thawing. The freeze–thaw resistance is strongly influenced by the properties of air voids in concrete as well as its content [19]. Mindess et al. [24] reported that the spacing factor should not exceed 0.2 mm to ensure adequate freeze-thaw protection. In addition to the spacing factor, specific surface area is also an important factor for a protective air void system. The air void parameters measured by AVA are tabulated in Table 3. Superplasticizers may occasionally show good freezing and thawing resistance even when the air void parameter limits are exceeded; however, it can be clearly confirmed that N-SRA improves the air void system (Table 3).

## 4. Discussion

It is shown that a very small dosage of C-SRA or N-SRA (the dosage of N-SRA being half the amount compared to C-SRA) reduces shrinkage and shows a high resistance of shrinkage-induced cracking under restrained conditions. Various mechanisms have been proposed for shrinkage reduction by SRA, including lowering of the surface tension of the cement paste pore solution and reduction of capillary stress [2,3,4,5,6,7,8,9]. The water in the capillary pores acts as bulk water to create menisci. However, when the capillary pores are fully occupied with water or when they are fully dried, menisci are not created.

The shrinkage reduction in C-SRA can be explained by the reduction in surface tension. C-SRA is composed of an assembly of ethylene oxide (EO) chains, which show hydrophilicity, and hydrophobic part of methyl group or propylene oxide (PO). Therefore, C-SRA has surface-active properties that can decrease the surface tension in the capillary pores. The determined R_H_ value of C-SRA is 459 nm (higher than 10 nm) in the synthetic cement pore solution, which indicates that, C-SRA, when used as a surfactant, easily forms micelle in vain. It is suggested that the ineffectiveness in shrinkage reduction by creating micelles results in a higher dosage of C-SRA.

It is believed that the calcium silicate hydrate (C-S-H) microstructure affects the performance of N-SRA in concrete. Jennings highlighted that the colloidal behaviour of C-S-H influences the shrinkage strain [25,26]. C-S-H is composed of calcium silicate sheets separated by interlayer spaces filled with water and calcium ions. The space between the calcium silicate sheets is the interlayer porosity of C-S-H. In addition, the C-S-H contains gel pores (1–10 nm) and isolated capillary pores (larger than 10 nm). The interlayer spaces in the C-S-H gel contain water that is removed during drying and re-enters during rewetting, which causes shrinkage and swelling [25,26]. In the gel pores, the surfaces are too close to each other, which restricts menisci formation. The determined R_H_ value of N-SRA, which is a water-soluble compound, was 9 nm in the synthetic cement pore solution, and thus it can be easily stored in the gel pore (1–10 nm). It is well known that a hydrophilic compound can hold water molecules through interactions [27,28]. Therefore, it is suggested that N-SRA molecules are stored inside the gel pores to prevent the evaporation of capillary water, where N-SRA serves as a water-retainer. N-SRA does not create micelles in the capillary pores due to sufficient hydrophilicity, which is also one of the reasons why effective for shrinkage reduction is observed in Concrete-N even with a small dosage of N-SRA.

## 5. Conclusions

This study focused on the effect of a newly developed shrinkage-reducing agent (N-SRA) to mitigate the drying shrinkage, and the results were compared with those of a conventional shrinkage-reducing agent (C-SRA). N-SRA is a water-soluble compound, which helps N-SRA to act by a mechanism different from that of C-SRA. The measured and predicted surface tension of N-SRA is higher than that of C-SRA. Addition of SRA did not change seven-day compressive strength but reduced the early splitting tensile strength. Both SRAs induced strength development at a later age. The same degree of shrinkage reduction (approximately 50% reduction) was observed in both SRAs, where the dosage of N-SRA was half the amount of C-SRA. Furthermore, N-SRA significantly influences the occurrence of shrinkage cracking, that is, it withstands cracking for a longer period than the concrete with C-SRA or without SRA. With respect to durability, the addition of N-SRA significantly improves the freezing and thawing resistance due to the creation of a proper air void system.

## Figures and Tables

**Table 1:** Chemical composition and physical property of cement.

Mineral Composition	(%)
C_3_S	56
C_2_S	18
C_3_A	9
C_4_AF	9
Loss on ignition	1.91
Specific surface area	3300 cm^2^/g
Density	3.16 g/cm^3^

**Table 2:** Proportions, constituents, and properties of concrete mixtures.

	Reference	Concrete-N	Concrete-C
Coarse aggregate (kg/m^3^)	939	939	939
Fine aggregate (kg/m^3^)	853	853	853
Cement (kg/m^3^)	309	309	309
Water (kg/m^3^)	170	170	170
Superplasticizer (% BWOC)	0.80	0.30	0.80
N-SRA (% BWOC)	0	1.0	0
C-SRA (% BWOC)	0	0	2.0
w/c	0.55	0.55	0.55
Slump (cm)	19.0	18.5	19.0
Slump flow (cm)	290	285	295
Air (%)	5.5	5.4	4.9
Spacing factor (μm)	316	283	439
Setting time: Initial (h)	6:00	6:45	6:40
Final (h)	8:15	9:15	9:15

**Table 3:** Entrained air void system measured by air void analyser (AVA) for concrete with N-SRA, with C-SRA and without shrinkage-reducing agent (SRA).

Concrete Mix	SRA	Spacing Factor (μm)	Specific Surface Area (mm^2^/mm^3^)
Concrete-N	N-SRA	283	16.8
Concrete-C	C-SRA	439	13.9
Reference	-	316	15.6
